# Sensory Perception and Consumer Acceptance of Carrot Cultivars Are Influenced by Their Metabolic Profiles for Volatile and Non-Volatile Organic Compounds

**DOI:** 10.3390/foods12244389

**Published:** 2023-12-06

**Authors:** Thomas Nothnagel, Detlef Ulrich, Frank Dunemann, Holger Budahn

**Affiliations:** 1 Julius Kühn-Institut, Federal Research Centre for Cultivated Plants, Institute for Breeding Research on Horticultural Crops, Erwin-Baur-Str. 27, D-06484 Quedlinburg, Germany; thomas.nothnagel@julius-kuehn.de (T.N.);; 2 Julius Kühn-Institut, Federal Research Centre for Cultivated Plants, Institute for Ecological Chemistry, Plant Analysis and Stored Product Protection, Königin-Luise-Str. 19, D-14195 Berlin, Germany

**Keywords:** *Daucus carota*, volatile organic compounds, terpenes, polyacetylenes, phenylpropanoids, GC, HPLC, consumer preference decision

## Abstract

Sensory parameters as well as the volatile and non-volatile compound profiles of sixteen carrot cultivars were recorded to obtain insight into consumer preference decisions. The sensory test was carried out with a consumer panel of 88 untrained testers allowing a clear acceptance-based differentiation of the cultivars. Five individual sensory characters (sweetness, overall aroma, bitterness, astringency and off-flavor) supported this discrimination. Chemical analyses of volatile organic compounds, polyacetylenes, phenylpropanoids and sugars enabled us to correlate the influence of these ingredients on sensory perception. Higher concentrations of α-pinene, hexanal, styrene and acetophenone correlated with a better acceptance, as well as sweetness and overall aroma perception. In contrast, a low acceptance as well as a stronger perception of bitterness, astringency and off-flavor correlated with enhanced concentrations of camphene, bornylacetate, borneol, myristicine, falcarindiol, falcarindiol-3-acetate, laserin and epilaserin. The present study should support the development of new breeding strategies for carrot cultivars that better satisfy consumer demands.

## 1. Introduction

Carrots are among the most popular vegetable crops worldwide due to their outstanding flavor and high content of such health-promoting phytochemicals as carotenoids, anthocyanins, polyacetylenes and terpenes [1]. Consumers favor carrots for their typical aromatic and sweet notes. On the other hand, a bitter off-taste is the main reason for consumer complaints and a major problem for carrot processors [2]. The range of substances contributing to flavor includes those that interact with taste receptors and those that are volatile and interact with the receptors of the orthogonal and retronasal olfactory system [3]. Nevertheless, for the complex sensory perception, visual impression and texture are also critical [4,5].

The compounds described as most important for carrot flavor thus far sugars, bitter substances and terpenes. The main sugars associated with the sweetness perception are glucose, fructose and sucrose, which account for more than 95% of the free sugars and 40–60% of the stored carbohydrates in the carrot root [6]. Bitterness is a very complex trait in carrots, because numerous chemical compounds are potentially bitter: e.g., polyacetylenes, mono- and sesquiterpenes, phenylpropanoids and isocoumarins [2,7]. The presence of excessive amounts of falcarinol-type polyacetylenes, especially falcarindiol, has been demonstrated to contribute to the undesirable bitter taste of carrots and their products [8,9]. Also, phenylpropanoids like laserine and epilaserine were shown to have low bitter recognition thresholds [10]. The typical aroma of carrots arises mainly from volatile mono- and sesquiterpenes, representing approximately 98% of the volatile organic compounds (VOCs) [11]. Some studies have been focused on the correlation between volatile terpenes and carrot sensory attributes [12,13,14]. Because carrots contain a complex blend of many different terpenes, it is a challenging task to relate individual terpenes to specific aroma notes. Flavor-associated volatiles are normally present in carrot roots at very low concentrations and are comparatively difficult to quantify. Thus, these compounds have been ignored in the breeding process for a long time.

During the past few decades, substantial progress has been achieved in quantifying potential carrot flavor compounds with analytical methods such as gas chromatography (GC) and high-performance liquid chromatography (HPLC). Kjeldsen et al. [15] established a link between the carrot aroma and certain isolated terpenes by a GC-olfactometry (GC-O) approach. The availability of a high-quality carrot reference genome sequence [16] has enabled ‘multiomics’ approaches. In combination with transgenic and classical genetic tools, these advances now make it far easier to identify key phytochemicals with a decisive influence on carrot flavor and liking scores [3,17,18,19,20,21,22].

Successful applications of such combined approaches have been shown especially for tomato and strawberry. Modern tomato cultivars have lost important flavor compounds still present in older cultivars and wild relatives. In the past few decades, many scientific studies have investigated the genetic components of tomato flavor in modern tomato cultivars and their relatives, including SNP-based GWAS (genome-wide association study) and candidate gene identification (for review, see [23]). To understand which substances have been lost during tomato breeding, Tieman et al. [24] used a 100-person consumer panel over five years to evaluate 150 tomato accessions and identified a set of sugars, organic acids and 29 volatiles that correlated positively or negatively with flavor and acceptance. Similarly, in strawberry, thirty volatile compounds have been stated to be positively and one negatively correlated to flavor intensity. Further analysis identified individual volatile compounds that have an enhancing effect on the perceived sweetness intensity of the fruit, independent of sugar content [25]. However, in this study no compounds have been found that directly correlated with consumer acceptance. In contrast, Ulrich and Olbricht [26] found positive correlations between consumer acceptance and the perception of sweet aroma intensity in strawberry. This result was confirmed by Fan et al. [27] who showed that consumer preference is highly related to the components of sweetness, flavor intensity and texture.

Compared to the advanced studies in tomato, strawberry, blueberry [28,29] and apple [30,31], less sensory research has been focused thus far on carrots. Rosenfeld et al. [32] investigated the influence of cultivation conditions (temperature and plant density) on volatile terpene and sugar levels and on sensory attributes of carrot roots by using a panel of ten trained testers. They concluded that several terpenes were responsible for the bitter taste and suppressed the perception of the sweet taste in carrots. Twelve trained panelists evaluated sensory quality parameters including the sweetness and off-flavor of differently colored carrots stored under a controlled atmosphere [33]. Based on a panel of eight trained persons, Fukuda et al. [14] investigated the aroma characteristics and volatile profiles of fourteen carrot cultivars belonging to the Kuroda and Flakee types. The quantitative contributions of terpenes including bisabolene isomers to ink-like, harsh and fruity notes were demonstrated, and it was concluded that the great diversity of terpene volatiles affects carrot aroma traits [14].

The improvement of flavor by traditional breeding is complicated due to strong environmental effects on the complex and diverse set of phytochemicals mediating flavor perception. Furthermore, there exist negative associations between flavor components and grower-demanded agronomic traits [27]. Therefore, breeding activities on sensory traits are more or less at a provisional level for the majority of cultivated plant species. The aim of the present study was to link sensory data based on a consumer acceptance test and comprehensive analytical datasets for volatile organic compounds, polyacetylenes, laserines and sugars to identify phytochemicals with significant a positive or negative impact on consumer preference in carrots.

## 2. Materials and Methods

### 2.1. Plant Material

Sixteen orange-colored carrot (*Daucus carota* L.) cultivars were selected on the basis of geographic origin and genetic distance [17,34]. The cultivar specifications are listed in Table S1. Carrot plants were grown on the JKI experimental field in Quedlinburg (51°47′ N, 11°8′ E, North Harz Foreland Region, Loess-black soil (Lö 1a), altitude: 140 m a.s.l., long year average of annual rainfall: 497 mm, long year average temperature: 8.9 °C, preceding crop: green oat). Plots (3 m × 1.5 m) were arranged in a blockwise randomized design with four biological replications. The seeds were sown in flat beds using a seed drill. Two rows with a distance of 45 cm were drilled per plot at a density of 100 seeds/m. Approximately 10 L/m^2^ water (sprinkler irrigation) was given twice to avoid seasonal drought stress. No fertilizer, fungicides or pesticides were applied. Carrots were harvested manually 120 days after sowing. After washing all visually healthy and undamaged roots by hand, 2 cm of the root tips and shoulders of each root was cut off and discarded. The remaining middle part of the roots was diced into pieces of about 10 × 10 × 10 mm. Aliquots of the root material (100 g per plot) were immediately frozen in liquid nitrogen and stored at −80 °C for chemical analyses. For sensory analyses, the diced root material of replicate plots was pooled for the consumer acceptance test.

### 2.2. Consumer Acceptance Test and Panel Structure

A total of 88 untrained adult testers participated in the two-day carrot sensory study. The panel consisted of 65 female and 23 male testers between 20 and 73 years old. Seven testers (8%), five females and two males, were smokers. Furthermore, the testers were asked for profession and hobbies. In total, sixteen carrot cultivars were tested, eight at the first, and a further eight on the second of two consecutive days. A sensory protocol was used, which was developed by Hoberg and Ulrich (JKI, unpublished, Figure S1). Directly before the test, all testers were instructed by the investigator. The carrot pieces were presented anonymously in plastic beakers. The testers picked up the carrot pieces with a spoon and first assessed the parameter acceptance. Thereafter, the five individual sensory characteristics of sweetness, aroma, bitterness, astringency and off-flavor (untypical for carrot) were assessed as specified in the template sheet (Figure S1). Water was used to rinse the mouth between samples.

### 2.3. Analyses of Volatiles

The main difference between both of the used analytical methods is the used sorptive sampling method. While in the HS-SPME technique [35] a coated fiber is used for the adsorption of VOCs from the head space of the vial, in the second enrichment technique (commercialized under the name Twister^TM^) [36] a coated stir bar serves as a target for volatile and semi-volatile compounds from the liquid phase.

#### 2.3.1. Volatile Analysis by HS-SPME-GC-FID

To prepare an enzyme-inhibited homogenate, 50 g aliquots of diced root material were thawed and homogenized in 150 g of a 20% NaCl solution (*w*/*v*) for 1 min using a Waring Blender. Afterwards, the resulting mixture was filtered using a 200 µm mesh gauze. For each sample, four 20 mL headspace vials each containing 4 g solid NaCl for saturation were filled with a 10 mL aliquot of the supernatant. After the addition of 10 μL internal standard solution (IST, 1-decanol in ethanol, c = 0.0005), the vials were sealed with a magnetic crimp cap including a septum.

For automated HS-SPME-GC-FID, a 100 μm polydimethylsiloxane fiber (Supelco, Bellefonte, PA, USA) and an MPS2-autosampler (Gerstel, Mühlheim an der Ruhr, Germany) were used. After equilibration for 10 min at 35 °C using a shaker (300 rpm), the fiber was exposed to the headspace for 15 min at 35 °C. Desorption was performed within 2 min in splitless mode and 3 min in split mode at 250 °C for thermal cleaning of the fiber.

A 6890 GC System (Agilent Technologies, Santa Clara, CA, USA) equipped with a HP-5ms column (0.25 mm inner diameter, 30 m length and 0.5 μm film thickness) and an FID were used for separation and detection. Hydrogen was used as carrier gas at a flow rate of 1.1 mL/min. The following temperature profile was applied: 45 °C (held for 5 min), from 45 to 210 °C at 3 K/min, 210 °C (held for 25.5 min). The volatiles were identified by parallel analyses of selected samples on an identically equipped GC-MS system. Detected compounds were annotated on the basis of library searches (NIST and MassFinder), retention indices and co-elution of authentic reference compounds (except for germacrene). For each carrot cultivar, four agronomical replicates (plots) were analyzed with two analytical replications.

#### 2.3.2. Volatile Analysis by SBSE-GC-MS

An 8 mL aliquot of the NaCl saturated homogenate (see Section 2.3.1) was transferred without any solid NaCl in an empty glass vial for volatile isolation by SBSE. A stir bar with a 0.5 mm film thickness and 10 mm in length coated with polydimethylsiloxan (PDMS) was placed in the liquid (Gerstel). The stir bar was used at 350 rpm and room temperature for 45 min. After removal from the carrot juice, the stir bar was rinsed with purified water, dried gently with a lint-free tissue and then transferred into a glass tube for thermal desorption and subsequent GC-MS analysis.

The parameters for the thermal desorption unit (TDU, Gerstel) and the cold injection system (CIS4, Gerstel) were the following: thermal desorption at 250 °C, cryo trapping at −150 °C. The TDU-CIS4 unit was used in Gerstel-modus 3: TDU splitless and CIS4 with a 15 mL/min split flow. The analyses were performed with an Agilent Technologies, 6890 N gas chromatograph equipped with an Agilent Technologies, 5975 B quadrupole MS detector. Compounds were separated on a ZB-WAX plus column (0.25 mm inner diameter, 30 m length and 0.5 μm film thickness). Helium was used as a carrier gas with a column flow rate of 1.1 mL/min. The following temperature program was used: 45 °C (held for 3 min), temperature gradient 3 K/min to 210 °C, 210 °C (held for 30 min). The mass spectrometer was used with electron ionization at 70 eV in the full scan mode. All individual plot samples were run with two analytical repetitions from an identical part of the supernatant.

#### 2.3.3. Data Processing for GC Analyses

The commercial software ChromStat version 2.6 by (Analyt-MTC GmbH, Müllheim, Germany) was used for raw data processing. The data inputs for ChromStat 2.6 were raw data from the percentage reports (retention time/peak area data pairs) performed with the software package Chemstation (version Rev.B.02.01.-SR1) by Agilent Technologies. Using ChromStat version 2.6, the chromatograms were divided into up to 200 time intervals, each representing a peak (substance) occurring in at least one chromatogram of the analysis set. The peak detection threshold was set on the 10-fold value of noise. The values are given as raw data (peak area in counts) which can also be described as relative concentrations because of the normalized sample preparation as described above. The volatiles were identified by library search (l) (NIST and MassFinder), retention indices (r) and the co-elution (c) of authentic samples (Table 1).

### 2.4. Analysis of Non-Volatile Metabolites

Deep-frozen root material (ca. 100–120 g, pool of agronomical replicates) was freeze-dried on initially pre-cooled (−30 °C) plates for 7 days using a freeze-dryer (Gamma 1-16 LSC, condenser temperature −50 °C, pressure 0.04 mbar, Martin Christ Gefriertrocknungsanlagen GmbH; Osterode am Harz, Germany). Dried root material was homogenized using a tube mill (2 × 30 s, 25,000/min, IKA- Werke GmbH, Staufen, Germany) and stored at −80 °C until extraction.

#### 2.4.1. Analysis of Sugars

Dry root homogenate (25 ± 1 mg) was weighed into a 2 mL polypropylene tube and spiked with 65 µL of a solution of maltose monohydrate (50 g/L) in acetonitrile/water, 1/1 (*v*/*v*). After the addition of 950 µL acetonitrile/water, 1/1 (*v*/*v*), the mixture was sonicated (10 min, 20 °C) and incubated on an overhead shaker (10 min, 20/min). After centrifugation (10 min, 13,000× *g*, 20 °C), the supernatant was transferred into a 2 mL volumetric flask. The residue was extracted once again with 950 µL acetonitrile/water, 1/1 (*v*/*v*) as described above. The volume of the combined supernatants was adjusted to 2 mL using acetonitrile/water, 1/1 (*v*/*v*). Afterwards, an aliquot of this solution was transferred into a 1.5 mL polypropylene tube and centrifuged (5 min, 13,000× *g*, 20 °C). The resulting supernatant was transferred into an HPLC vial and stored at room temperature until analysis.

Sugar analyses were performed on a 1100 Series HPLC system (Agilent Technologies) comprising a degasser (G1322A), a binary pump (G1312A), an autosampler (G1329A), an autosampler thermostat (G1330A), a column compartment (G1316A) and an evaporative light scattering detector (SEDEX 75, Sedere, Olivet, France). Extracts (method A, injection volume 10 µL) were separated on a Supelcosil LC-NH_2_ column (4.6 × 250 mm, 5 µm particle size, Sigma-Aldrich) by isocratic elution using acetonitrile/water, 8/2 (*v*/*v*) at a flow rate of 2 mL/min. The column temperature was maintained at 40 °C and the autosampler temperature at 20 °C. The total run time was 10 min. fructose (tR = 3.08 min), glucose (tR = 3.43 min), sucrose (tR = 4.83 min) and the internal standard maltose (tR = 5.42 min) were quantified based on peak area using individual external standard calibration curves (calibration range 5–50 µg on column, four levels with two technical replicates, calibration model lny = lna + blnx, R2 > 0.998 for each sugar). Sugar levels were corrected using the recovery rate of the internal standard maltose. Each of the sixteen pooled carrot samples was analyzed in triplicate (technical replicates).

#### 2.4.2. Analyses of Polyacetylenes and Laserines

Dry root homogenate (25 ± 1 mg) was weighed into a 2 mL polypropylene tube and spiked with 50 µL of a solution of nonivamide (200 mg/L) in methanol. After addition of 200 µL methanol and 500 µL chloroform, the mixture was shaken (10 min, 2400/min, room temperature) and sonicated (10 min, 20 °C). Afterwards, 250 µL TRIS buffer (50 mM, pH 7.5) was added. The mixture was shaken (2 min, 2400/min, room temperature) and centrifuged for phase separation (10 min, 13,000× *g*, 20 °C). The organic phase was carefully transferred into a new 2 mL polypropylene tube and the remaining mixture was re-extracted with 500 µL chloroform. The combined organic extracts were evaporated to dryness using a rotational vacuum concentrator (30 °C, 4 mbar). To the remaining residue, 250 µL acetone was added. The resulting mixture was shaken (2 min, 2400/min, room temperature), sonicated (3 min, 20 °C) and centrifuged (5 min, 13,000× *g*, 20 °C). The resulting supernatant was transferred into an HPLC vial and stored at 6 °C until analysis.

Analyses were performed on a 1100 Series HPLC system (Agilent Technologies) comprising a degasser (G1322A), a binary pump (G1312A), an autosampler (G1329A), an autosampler thermostat (G1330A), a column compartment (G1316A) and a diode array detector (G1315A). Extracts (method B, injection volume 5 µL) were separated on a Zorbax Eclipse XDB-C18 column (3.0 150 mm, particle size 3.5 µm, Agilent Technologies) using water and acetonitrile as eluent A and B, respectively. The following binary gradient program at a flow rate of 1 mL/min was used: 0–11.5 min, linear from 50 to 73% B; 11.5–12.0 min, linear from 73 to 100% B; 12.0–17.0 min, isocratic, 100% B; 17.0–20.0 min, isocratic, 50% B. The column and the autosampler temperature were maintained at 40 °C and 6 °C, respectively. The diode array detector response time was set at 0.2 s, the optical slit width at 4 nm. Polyacetylenes were detected at 196 nm, nonivamide at 204 nm and laserines at 225 nm with a spectral bandwidth of 4 nm. Nonivamide (tR = 2.5 min), falcarindiol (tR = 5.3 min), laserin (tR = 7.7 min), epilaserin (tR = 8.1 min), falcarindiol-3-acetate (tR = 9.0 min) and falcarinol (tr = 10.9 min) were quantified based on peak area using individual external standard calibration curves (for nonivamide: calibration range 1–600 ng on column, 14 levels, calibration model y = mx, equal weighting, R2 = 0.9998; for falcarindiol, falcarinol, laserin and epilaserin: calibration range 1–1000 ng on column, 12 levels, calibration model y = mx, equal weighting, R2 > 0.9999). Falcarindiol-3-acetate was quantified using the calibration curve of falcarindiol. Analyte levels were corrected using the recovery rate of the internal standard nonivamide. Each of the sixteen pooled carrot samples was analyzed in triplicate (technical replicates).

### 2.5. Statistics

Descriptive statistics (mean ± standard deviation) and one-way ANOVA followed by Tukey’s test were performed using Statistica version 7.1 (StatSoft Europe GmbH, Hamburg, Germany). The correlation analysis as well as principal component analysis (PCA) between the mean values of the sensory and analytic data were performed by the same software package. The heat map of the sensory analysis was constructed with the Systat version 13 (Systat Software, Inc., Chicago, IL, USA).

## 3. Results

### 3.1. Consumer Acceptance Study

**Figure 1:**
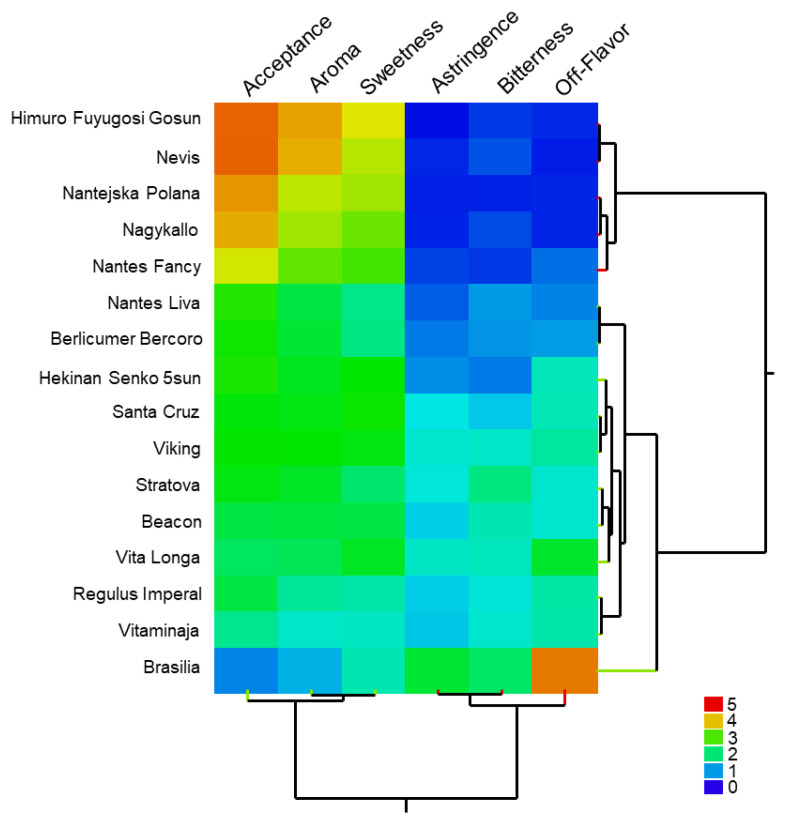
Heatmap of the sensory analysis (n = 88 testers, hierarchical clustering, Euclidian distance, single linkage method (nearest neighbor)).

No significant influences of age, gender, profession or hobbies on cultivar acceptance have been observed (results not shown). The sensory test showed a broad variation for all requested parameters and allowed a distinct differentiation of the sixteen cultivars (Figure 1). Four cultivars, ‘Nevis’, ‘Himuro Fuyugosi Gosun’, ‘Nagykallo’ and ‘Nantejska Polana’ were rated with high (>4.0) acceptance and ten with a good (3.7–2.7) or medium (2.6–2.1) acceptance. For two cultivars a poor acceptance (<2.0) was determined, with 1.8 for ‘Vitaminaja’ and 0.9 for ‘Brasilia’ as the worst cultivar under the given cultivation conditions (Table S2).

The sensory classification according to the five additionally assessed characters showed that cultivars rated with a high acceptance tended to be assessed as sweeter and expressed a typical carrot aroma. Furthermore, they were rated as less bitter, non-astringent and lacking off-flavor characteristics. In contrast, both cultivars rated with a low acceptance were assessed by a majority of testers as bitter and/or astringent, partially with off-flavors. For the latter ones, some testers stated chemical, soapy, musty, earthy or woody notes. The hierarchical cluster analysis using all sensory parameters revealed two main clusters, with ‘Brasilia’ clearly delineated in the lower cluster (Figure 1). While acceptance as well as sweetness and aroma correlate very strongly, there is a negative correlation between these and the parameters of bitterness, astringency and off-flavor.

### 3.2. Analyses of Volatile Organic Compounds Using Two Different Sampling Methods

Volatile profiles of the carrot cultivars were prepared applying both HS-SPME and SBSE sampling technologies (Table 1). In total, 94 peaks were detected by SPME and 199 peaks by SBSE technology, thereof 38 and 39 peaks were chemically identified, respectively. Seventeen of the identified substances were detected by both technologies. Of these, thirteen datasets were highly correlated (r = 0.78–0.98) (Table 1). Predominately, mono- and sesquiterpenes were detected and identified with the SPME approach (28 compounds) and the SBSE approach (12 compounds) of which 9 were detected with both techniques (Table 2). Twenty-five of the identified substances belong to alcohol-, aldehyde-, ketone-, glucosinolate-, carotenoid-, alcane- or phenylpropene pathways (Table 1). ANOVA and Tukey’s procedure showed significant differences between the tested cultivars for all of the identified substances (Table S2). For fifteen of the identified substances, only some of the cultivars exhibited values above the detection limit. For the other cultivars, zero values were noted (Table S2). Allocimen and (E)-ocimene were detectable in three and six cultivars, respectively, with ‘Viking’ outstanding due to its extremely high concentration for both. Myristicin was only detectable in two and seven cultivars, respectively, by SPME and SBSE extraction. For this substance, ‘Brasilia’ showed extremely high values in comparison to the other cultivars. The highest total VOC concentrations were measured for ‘Stratova’ using both SPME and SBSE techniques. Moreover, ‘Stratova’ showed the highest concentrations for 10 and 17 individual substances detected by SPME and SBSE, respectively. For terpinolene, 1,3,8-p-menthatriene, 2-methylcoumarine, dimethylstyrole, (E)-linaloloxide and p-methylacetophenone concentrations were measured that were two to four times higher than those of the other cultivars. Also, β-myrcene, limonene, γ-cadinene, p-methylacetophenone and p-cymen-8-ol were measured in ‘Stratova’ with significantly higher contents (Table S2). The lowest total VOC concentrations were detected for ‘Hekinan Senko 5sun’ by the SPME analysis and for ‘Vitaminaja’ by SBSE analysis.

The cultivars with the best ratings in terms of acceptance showed high values for 2-phenoxyethanol, nonanoic acid (‘Nevis’), α-pinene, styrene, elemicine (‘Himuro Fuyugosi Gosun’), and β-pinene (‘Nagykallo’), as well as β-caryophyllene and 1,2-cyclopentadione (‘Nantejska Polana’). The cultivar ‘Brasilia’, which was rated very negatively in acceptance, showed significantly higher values for camphene, o-cymene, dimethylstyrene, ß-sesquiphellandrene 1, borneol and benzyl alcohol compared to the other cultivars. In addition, the content of bornylacetate was more than tenfold higher. The cv. ‘Beacon’ also showed a striking VOC pattern with significantly higher levels of sabinene, 6-methyl-5-heptene-2-one, bergamethene, α-santalene, ß-sesquiphellandrene 2, (Z)-ß-farnesene and geranylacetone (Table 1 and Table S2). Among the 17 parallel datasets, the data with higher numerical values were used for correlation analyses (Table 1, Table 2 and Table S2).

**Table 1:** List of VOCs detected by HS-SPME-GC-FID and SBSE-GC-MS in a parallel approach.

Identified Substance	CAS No	Substance Group	SI	RT-	HS-SPME-GC-FID (A)				RT-	SBSE-GC-MS (B)				Corr
				SPME	MW ± SD	Min	Max	N	SBSE	MW ± SD	Min	Max	N	A:B
α-pinene	80-56-8	monoterpene	l r c	6.89	119.62 ± 72.74	28.74	264.97	16	7.30	5.73 ± 1.95	3.03	9.92	16	0.847
camphene	79-92-5	monoterpene	l r c	8.32	8.16 ± 9.19	0	39.51	15		n.i.				
undecane	1120-21-4	alcane	l r c	8.95	5.11 ± 7.78	0	27.86	11		n.i.				
hexanal	66-25-1	aldehyde	l r c	9.07	0.42 ± 1.38	0	5.47	2	9.68	8.73 ± 3.18	5.22	16.42	16	−0.190
β-pinene	127-91-3	monoterpene	l r c	9.58	84.71 ± 92.69	0	351.30	15	10.16	3.29 ± 4.67	0	17.12	13	0.969
sabinene	3387-41-5	monoterpene	l r c	10.41	37.52 ± 69.55	0	281.11	13	10.73	5.13 ± 6.51	0	26.15	12	0.929
β-myrcene	123-35-3	monoterpene	l r c	11.75	194.91 ± 186.08	45.91	648.29	16	12.79	5.36 ± 6.25	0	19.19	15	0.984
limonene	138-86-3	monoterpene	l r c		n.i.				14.09	9.11 ± 2.95	4.83	17.94	16	
β-phellandrene	555-10-2	monoterpene	l r c		n.i.				14.83	0.14 ± 0.25	0	0.73	4	
limonene + b-phellandrene	mixture	monoterpenes	l r c	13.11	129.33 ± 71.91	51.31	356.61	16		n.i.				
(*E*)-ocimene	3779-61-1	monoterpene	l r c	14.17	21.92 ± 60.92	0	236.53	6		n.i.				
(*E*)-2-hexenal	6728-26-3	aldehyde	l r c		n.i.				15.76	6.32 ± 2.06	3.91	10.88	16	
γ-terpinene	99-85-4	monoterpene	l r c	14.57	146.03 ± 89.81	13.93	327.70	16	16.63	3.87 ± 2.76	0	9.06	14	0.929
(*Z*)-ocimene	3338-55-4	monoterpene	l r c	14.76	14.50 ± 20.10	0	67.69	9		n.i.				
styrene	100-42-5	styrol	l r c		n.i.				17.45	2.04 ± 0.33	1.23	2.57	16	
o-cymene	527-84-4	monoterpene	l r c	15.40	65.98 ± 32.49	15.14	119.86	16	18.02	10.38 ± 6.94	2.76	25.61	16	0.430
terpinolene	586-62-9	monoterpene	l r c	15.89	1004.40 ± 479.59	509.32	2471.94	16	18.65	75.55 ± 32.70	46.62	181.10	16	0.932
6-methyl-5-heptene-2-one	100-93-0	ketone	l r c	17.58	3.60 ± 5.76	0	18.86	10	21.16	24.76 ± 20.55	7.15	76.00	16	0.125
hexanol	111-27-3	alcohol	l r c		n.i.				21.86	3.16 ± 1.08	1.45	5.17	16	
allocimene	3016-19-1	monoterpene	l r c	18.58	5.64 ± 16.85	0	65.67	3		n.i.				
1,3,8-p-menthatriene	18368-95-1	monoterpene	l r	19.30	10.11 ± 5.97	4.08	29.39	16	23.57	56.39 ± 35.30	28.41	170.33	16	0.972
2-methylcoumarin	92-48-8	coumarine derivate	l r	20.28	8.68 ± 5.35	3.31	26.44	16	25.07	46.51 ± 34.70	18.41	157.78	16	0.971
dimethylstyrol	1195-32-0	styrol	l r c	20.57	16.62 ± 7.74	5.64	40.22	16	25.51	47.52 ± 24.22	22.82	124.30	16	0.920
β-sesquiphellandrene 1	20307-83-9	sesquiterpene	l r c	21.39	10.60 ± 14.53	0	57.65	14		n.i.				
γ-cadinene	483-74-9	sesquiterpene	l r	22.10	6.35 ± 5.36	0	20.44	15		n.i.				
α-bergamotene	17699-05-7	sesquiterpene	l r	24.17	7.57 ± 17.23	0	67.14	9		n.i.				
α-santalene	512-61-8	sesquiterpene	l r	24.30	3.77 ± 6.38	0	24.83	9		n.i.				
(*E*)-linaloloxide	34995-77-2	monoterpene oxide	l r		n.i.				26.33	3.12 ± 3.00	0	10.45	14	
furfural	98-01-1	aldehyde	l r c		n.i.				26.90	17.99 ± 6.21	9.83	31.97	16	
2-ethylhexanol	104-76-7	alcohol	l r c		n.i.				27.72	6.51 ± 1.62	4.34	10.53	16	
benzaldehyde	100-52-7	aldehyde	l r c		n.i.				29.32	8.02 ± 1.48	5.30	11.21	16	
bornylacetate	76-49-3	monoterpene ester	l r c	24.67	136.00 ± 304.17	11.99	1266.76	16	30.47	9.48 ± 1.92	6.75	13.47	16	0.286
β-caryophyllene	87-44-5	sesquiterpene	l r c	25.09	697.97 ± 329.24	49.86	1123.34	16	31.89	11.26 ± 6.66	0.88	21.51	16	0.749
β-sesquiphellandrene 2	20307-83-9	sesquiterpene	l r	26.14	5.91 ± 6.34	0	26.86	16		n.i.				
(*Z*)-β-farnesene	28973-97-9	sesquiterpene	l r c	26.42	46.45 ± 60.38	6.93	245.64	16		n.i.				
humulene	6753-98-6	sesquiterpene	l r	26.94	56.63 ± 28.61	5.62	99.75	16		n.i.				
borneol	507-70-0	monoterpene alcohol	l r c	27.73	4.56 ± 15.18	0.00	61.24	5		n.i.				
germacrene	28387-44-2	sesquiterpene	l r c	27.95	40.19 ± 39.79	5.97	130.21	16		n.i.				
β-bisabolene	15352-77-9	sesquiterpene	l r c	28.16	39.48 ± 14.79	8.80	58.01	16		n.i.				
(*Z*)-α-bisabolene	495-62-5	sesquiterpene	l r	29.28	64.01 ± 43.36	3.47	139.59	16		n.i.				
geranylisobutyrate	2345-26-8	monoterpene ester	l r	30.09	1.77 ± 2.12	0	7.14	9		n.i.				
p-cymen-8-ol	1197-01-9	monoterpene alcohol	l r c	31.12	5.42 ± 3.23	0	13.61	15	41.59	128.22 ± 67.17	53.40	323.98	16	0.895
butyrolactone	96-48-0	lactone	l r c		n.i.				33.60	2.43 ± 0.84	0.67	4.00	16	
acetophenone	98-86-2	ketone	l r c		n.i.				34.46	351.22 ± 61.61	276.83	484.52	16	
furanmethanol	98-00-0	alkohol	l r c		n.i.				34.76	13.96 ± 2.11	11.23	19.00	16	
isothiocyanato cyclohexane	1122-82-3	alcane	l r		n.i.				35.07	3.31 ± 3.59	0.88	14.71	16	
phenyl-2-propanone	103-79-7	ketone	l r		n.i.				37.16	3.70 ± 4.50	0.42	17.94	16	
dextrocarvone	2244-16-8	monoterpene	l r		n.i.				37.47	6.80 ± 2.87	2.97	10.81	16	
1,2-cyclopentadione	3008-40-0	ketone	l r		n.i.				38.83	7.21 ± 1.73	5.40	10.67	16	
p-methylacetophenone	122-00-9	ketone	l r		n.i.				39.09	15.98 ± 8.78	7.05	45.21	16	
geranylacetone	3796-70-1	monoterpene ketone	l r	31.22	7.49 ± 6.52	0.00	25.50	14	41.84	12.18 ± 4.77	6.27	19.42	16	−0.094
geranylisovalerate	109-20-6	monoterpene ester	l r	32.07	13.91 ± 12.78	0.00	48.76	14		n.i.				
β-ionon	79-77-6	carotinoid	l r c	33.34	1.17 ± 1.99	0.00	6.58	7		n.i.				
caryophyllenoxid	1139-30-6	sesquiterpene	l r c	34.53	4.32 ± 3.53	0.00	13.50	14		n.i.				
benzylacohol	100-51-6	alcohole	l r c		n.i.				42.71	9.25 ± 5.86	3.36	24.59	16	
4-methylphenol	106-44-5	phenol	l r c		n.i.				49.54	4.02 ± 1.53	0.58	6.71	16	
2-phenoxyethanol	122-99-6	alcohole	l r c		n.i.				51.42	17.20 ± 3.90	8.12	23.00	16	
nonanoic acid	112-05-0	alcane acid	l r c		n.i.				52.39	11.25 ± 2.75	7.39	18.56	16	
elemicine	487-11-6	phenylpropanoid	l r		n.i.				54.08	16.00 ± 5.15	6.64	25.33	16	
myristicine	607-91-0	phenylpropanoid	l r c	40.18	2.71 ± 10.03	0.00	40.21	2	55.11	7.90 ± 27.12	0.00	109.29	7	0.999
Number					38					39				17

CAS—Chemical Abstracts Service; SI—substance identification l = library search, r = retention indices, c = co-elution; n.i.—concentration under the detection level; RT—retention time; MW—means; SD—standard deviation; Min—minimum value; Max—maximum value; N—number of cultivars where the substances were detected; Corr—correlation between both approaches.

### 3.3. HPLC Analyses of Polyacetylenes, Laserines and Sugars

The polyacetylenes falcarinol, falcarindiol and falcarindiol-3-acetate, the phenylpropanoids laserin and epilaserin, and the sugars fructose, glucose and sucrose were quantified for the root tissue of tested cultivars as summarized in Table 2 and Table 3. ANOVA and Tukey’s procedure showed significant differences between the cultivars. The falcarinol content varied between 132 and 463 µg/g DW (dry weight) for ‘Nantes Fancy’ and ‘Nantes Liva’, respectively. Cultivars with a high content of falcarinol tend to have lower falcarindiol and falcarindiol-3-acetate levels. The falcarindiol content varied from 128 µg/g DW for ‘Hekinan Senko’ to 560 µg/g DW in ‘Brasilia’, and falcarindiol-3-acetate varied from 20 to 113 µg/g DW in a similar cultivar order as falcarindiol. What is striking about the cv. ‘Brasilia’ are the extremely high contents of falcarindiol and falcarindiol-3-acetate, with 560 and 113 µg/g DW, respectively. In addition, a great variation was detected for the phenylpropanoids laserin and epilaserin, ranging between 0 and 521 and 0 and 572 µg/g DW, respectively. The extreme differences between the value of 0 (not detectable) in ‘Vitaminaja’ and over 500 µg/g DW in ‘Brasilia’ are quite surprising (Table 2). Furthermore, significant differences between the cultivars were also found for three major sugar compounds determined using the same root samples (Table 3).

**Table 2:** Analytic data of main polyacetylenes and phenylpropanoids in carrot root tissue (µg/g DW). The plot mean and standard deviation (SD) of the individual cultivars as well as ANOVA (*p*) and Tukey’s significance test are given (different letters suggest significance *p* = 0.01, cultivars sorted by acceptance).

Cultivar	Falcarinol	Falcarindiol	Falcarindiol-3-Acetate	Laserin	Epilaserin
	Mean ± SD	Mean ± SD	Mean ± SD	Mean ± SD	Mean ± SD
Nevis	349.47 ± 8.21 d	164.75 ± 2.84 i	20.63 ± 1.24 i	74.04 ±1.74 c	27.12 ± 0.39 c
Himuro Fuyugosi Gosun	178.93 ± 2.88 i	237.40 ± 3.08 efg	30.37 ± 0.76 gh	7.16 ±0.51 ij	14.11 ± 0.91 cd
Nantejska Polana	430. 44 ± 8.32 b	168.93 ±12.04 i	32.82 ± 1.29 gh	15.54 ± 0.20 fg	14.07 ± 0.42 cd
Nagykallo	231.39 ± 2.70 fg	333.90 ± 11.55 b	48.15 ± 0.95 e	22.26 ±0.98 f	20.79 ± 0.28 cd
Nantes Fancy	132.41 ± 1.80 k	201.01 ± 1.35 h	27.15 ± 0.75 h	9.81 ± 0.11 hij	4.19 ± 0.41 d
Nantes Liva	463.05 ± 18.35 a	255.30 ± 16.42 def	36.62 ± 2.75 fg	10.79 ± 0.40 hij	3.81 ± 0.47 de
Berlicumer Bercoro	256.57 ± 3.65 ef	167.49 ± 8.49 i	30.70 ± 0.88 gh	16.87 ± 0.81 fg	27.16 ± 0.94 c
Hekinan Senko 5sun	276.17 ± 6.45 e	128.93 ± 1.33 j	54.56 ± 0.77 d	13.91 ± 0.26 ghi	7.39 ± 0.05 d
Viking	166. 27 ± 3.34 ij	266.27 ± 5.73 cd	58.06 ± 1.16 d	19.84 ± 1.01 fg	30.31 ± 1.34 bc
Santa Cruz	345.64 ± 8.02 d	211.46 ± 4.77 gh	33.16 ± 1.43 gh	33.96 ± 2.09 e	29.75 ± 2.70 bc
Stratova	134.06 ± 2.32 k	329 ± 17.92 b	60.38 ± 3.20 d	84.02 ± 3.92 b	54.66 ± 2.46 b
Beacon	184.35 ± 7.82 hi	263.41 ± 9.27 de	83.90 ± 3.51 b	49.70 ± 1.90 d	46.47 ± 2.08 b
Regulus Imperial	208.33 ± 2.48 gh	232.06 ± 1.96 fg	72.88 ± 2.02 c	2.69 ± 0.26 j	4.53 ± 0.33 d
Vita Longa	243.60 ± 2.75 f	290.07 ± 5.65 c	33.17 ± 1.17 gh	43.35 ± 2.68 d	28.43 ± 0.95 bc
Vitaminaja	383.93 ± 11.97 c	196.24 ± 6.91 h	42.30 ± 0.80 ef	0.00 ±0.00	0.00 ±0.00
Brasilia	144.18 ± 1.89 jk	560.78 ± 3.25 a	113.16 ± 1.97 a	521.77 ± 6.91 a	572.22 ± 19.93 a
ANOVA (p)	0.00	0.00	0.00	0.00	0.00
Tukey p = 0.01					

### 3.4. Pearson Correlation Analysis and Principle Component Analysis (PCA)

A relatively high number of correlations were detected between the sensory parameters and the chemical compounds of GC- and HPLC analyses. High sensory acceptance as well as sweetness and typical carrot aroma correlated positively with the VOCs α-pinene, hexanal, ß-pinene, styrene, ß-caryophyllene and acetophenone. In contrast, camphene, 6-methyl-5-heptene-2-one, bornyacetate, borneol, dextrocarvone, ß-ionon, geranylacetone, benzylalcohol, 4-methylphenol and myristicin were highly correlated with the negative sensory characteristics of bitterness, astringency and off-flavor (other than carrot). Additionally, the polyacetylenes falcarindiol and falcarindiol-3-acetate as well as the isocoumarins laserin and epilaserin were highly correlated with the negative sensory characteristics of bitterness, astringency and off-flavor. Sweetness correlated positively with the content of sucrose (Table 4). A large number of correlations also exist between the individual substances but were not considered in detail in this study (Table S3).

**Figure 2:**
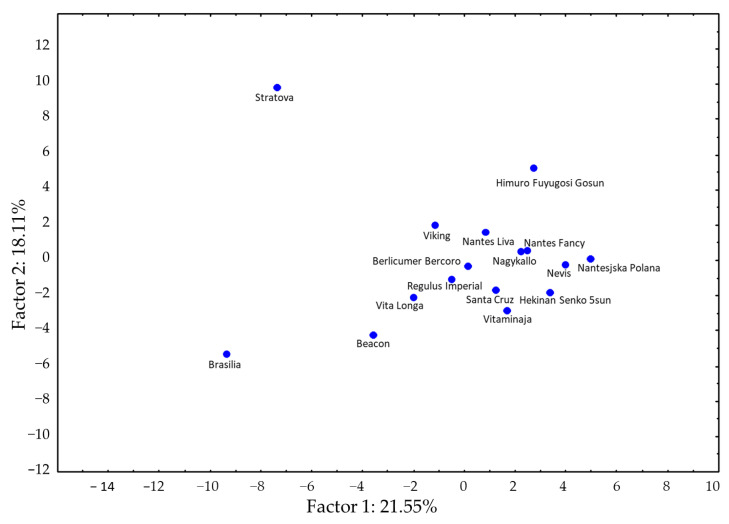
PCA score plot of 6 sensory parameters and 67 chemical substances of the sixteen carrot cultivars. Distribution from low to high acceptance on Axis 1 from left to right.

**Figure 3:**
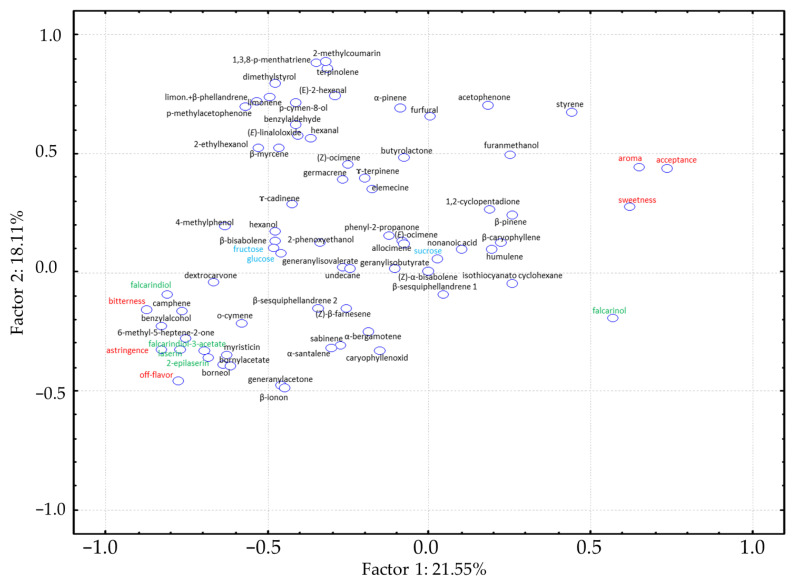
PCA loading plot of the sensory and instrumental attributes of the sixteen carrot cultivars (red—6 sensory attributes, black—59 volatile organic compounds, green—3 polyacetylenes and 2 laserins, blue—3 sugars).

The principal component analyses were performed on the basis of the sensory data (Table S2) and mean values of the concentration for 59 identified VOCs (Table 1 and Table S3), as well as three polyacetylenes, two laserins (Table 2) and three sugar compounds (Table 3). The cultivars are widely distributed in the parameter space and localized along Axis 1 for cultivars with low to high acceptance from left to right, respectively. The most accepted cultivars ‘Himuro Fuyugosi Gosun’, ‘Nantesjska Polana’ and ‘Nagykallo’ are located in the right upper quadrant, with ‘Nevis’ on the *x*-axis of the right side. Cultivars classified as poor or with off-flavor, e.g., ‘Brasilia’, ‘Beacon’, ‘Regulus Imperial’ or ‘Vita Longa’ are in the left lower quadrant, with ‘Brasilia’ clearly off in an outlier position. The cultivar ‘Stratova’, rated as medium acceptable, is also located in an outlier position in the left upper quadrant. For this cultivar, the highest total volatile content was measured, as well as significantly higher contents for 15 volatiles compared to the other cultivars. Cv. ‘Viking’, located in the same quadrant, showed a similar tendency (Figure 2). The corresponding loading plot (Figure 3) illustrates the associations between sensory and instrumental traits in the cultivars studied.

## 4. Discussion

Using the rapid progress of metabolome research, in the last twenty years, some key players impairing carrot taste and aroma have been identified [8,9,10]. A combination of a sensory tests and chemical analyses should provide the information, if qualitative and quantitative differences in the metabolome of the 16 tested carrot cultivars are relevant for the overall consumer acceptance and the perception of sensory key notes. Sensory tests can be performed by a relatively small, well-trained tester panel [14,32] or by a greater panel of untrained consumers (at least 80 persons) [37]. For the present study, 112 untrained testers have been registered. Finally, 88 (79%) of them took part on both days and provided completely filled protocols as the data basis for the association study.

High acceptance (>4.0) was assigned to four cultivars, ten cultivars were rated as good to moderate, and two cultivars rated poor to very poor. Almost all testers classified ‘Brasilia’ as the worst cultivar in the test set, mainly due to the strong bitterness and off-flavor notes (untypical for carrots). One reason for the very poor sensory performance of ‘Brasilia’ could be the fact that this cultivar was bred for tropical or subtropical regions and the Central European climatic conditions in Quedlinburg are probably suboptimal for this cultivar. Various studies have shown that in addition to genetic factors, specific environmental conditions such as waterlogging, drought, heat or cold stress, as well as mechanical stress (e.g., harvest or transport stress), can affect the accumulation of secondary metabolites and as a consequence also the sensory attributes [2,7,38,39,40,41]. Since in our study the growing and testing conditions were kept constant for all cultivars, genetic factors appear to be mainly responsible for the clear differences in acceptance and the five individual sensory characteristics between the tested cultivars.

The parallel usage of two independent analytical methods, HS-SPME-GC-FID and SBSE-GC-MS, for the detection of volatile and semi-volatile organic compounds provided a more detailed overview on the intermediates and final products of various metabolic pathways in carrot roots than the usage of a single method for VOC analysis. Seventeen metabolites were identified by both methods and for thirteen of them a remarkable high correlation between both methods (r = 0.78–0.98) can be stated. But 21 of the identified metabolites were exclusively detected using SPME extraction and 22 by SBSE enrichment. The high complementarity of both methods confirms our strategy to combine the metabolic profiles generated by the two different analytical methods. The combination of different sample preparation methods was already demonstrated for other foods [42]. The fiber used for HS-SPME is provided with only 0.6 µL of the adsorbent material (PDMS), whereas the volume of the absorbent phase for SBSE is up to the 200-fold, because of the thick sorption layer on the stir bar [36]. This increases the sorption potential and provides the chance to concentrate also minor substances to a level over the detection limit [43,44,45]. Otherwise, using the SBSE technique, there might be the risk for the binding and detection of unspecific products because of the direct contact of the stir bar and the liquid phase. Additionally, the higher number of peaks for the SBSE technique (199 vs. 94) could reduce the resolution.

The three measured sugars (glucose, fructose and sucrose) are expected to be highly correlated to sweetness. But in our study, the correlation of sucrose content with sweetness perception was calculated only at 0.444 and that for glucose and fructose was even lower (Table 4). Thus, further substances must be responsible for the sweetness sensation. In strawberry, sweetness perception and consumer acceptance were enhanced by various esters, aldehydes, lactones and ketones. For instance, γ-dodecalactone and γ-decalactone were able to increase sensory sweetness independent of sugars [27]. Some volatiles, which have been described with sweet aroma notes for carrot (terpinolene, limonene, β-bisabolene and β-ionene) [15], were not significantly correlated with sweetness sensation in our study. Otherwise, a positive impact on sweetness perception was confirmed for the typical carrot monoterpene α-pinene and the aldehyde hexanal. The enhancement of the sweetness sensation by styrene and acetophenone is a newly described phenomenon for carrot. Styrene, a styrol compound, first described for carrots by Alasalvar et al. [12] has a sweet smell, but higher concentrations can cause a less pleasant odor [46,47]. Higher concentrations of acetophenone might also contribute to off-odors, as reported for watermelon [48]. In blackberry, however, the ‘hawthorn note’ of acetophenone provides a very attractive top flavor [49].

Excessive amounts of falcarinol-type polyacetylenes have been reported to contribute to the undesirable bitter taste of carrots and their products such as juice or puree [9,50]. In our sensory study, falcarinol was not correlated with bitterness, whereas falcarindiol and especially its putative derivative falcarindiol-3-acetate were highly correlated with bitterness and off-flavor. Breeding approaches using molecular markers for key enzymes of polyacetylene synthesis might reduce unwanted polyacetylenes to an appropriate level and be helpful to improv consumer acceptance [21]. The highest concentrations by far for falcarindiol and falcarindiol-3-acetate were determined for the least appreciated cultivar ‘Brasilia’. This cultivar was also found to contain extremely high levels of laserine and epilaserine, which are also among the substances involved significantly in bitterness and off-flavor perception. Both phytochemicals belong to the group of phenylpropanoids.

Schmiech et al. [10] described, besides the above mentioned polyacetylenes and phenylpropanoids, a dozen other substances with bitter recognition thresholds below 50 µmol/kg. In our study, we identified additional phytochemicals, which have not been reported in other carrot bitter senso-metabolome studies before: the apocarotenoid 6-methyl-hepten-2-one and the monoterpene ketone dextrocarvone. The former is a product of carotenoid degradation and has been identified in ripe red tomatoes [51] and dark-rooted carrots [52]. In watermelon, this substance is the most abundant ketone in the fruit flesh and contributes to green, musty and fruit odors [53]. Dextrocarvone (D-carvone) is known for its spicy aroma with notes like caraway seeds [54], but its obvious strong impact on carrot bitterness perception has not been reported before.

The contents of camphene, borneol and bornylacetate in our study are strongly correlated with off-flavor notes. The highest concentrations by far of borneol and bornylacetate were found in ‘Brasilia’. Camphene showed a high correlation (r = 0.95) with its precursor borneol. The impression of these three compounds was described as camphoraceous by gas chromatography combined with olfactory analysis [55]. A camphoraceous scent is a strongly aromatic, almost medicinal scent [56]. The (too) high concentrations of borneol and its derivatives in carrots obviously play a major role in consumer disliking. Bornylacetate was found to be among the most abundant terpene compounds in carrot roots, and QTLs for this terpene and its putative precursor borneol have been identified on chromosome 2 of carrot [17]. Several other terpenes are involved in harsh or bitter flavor notes, and these off-flavor characteristics increase with terpene contents in different carrot genotypes [13]. Thus, the monoterpenes sabinene, α-terpinolene and β-pinene were predicted as candidates for bitterness in carrots [57]. But in our study, for sabinene and β-pinene, no effect on bitterness was observed.

Functional genomic analyses of key metabolites of carrot, including the components identified here, may provide marker tools for more efficient selection of desired genotypes with enhanced consumer preference. But it must be taken into account that some of the metabolites with bitter notes are also associated with fungal resistance [57,58,59], insect resistance [60] and tolerance to abiotic stress conditions [2,61].

In summary, the study showed that acceptance-based preference scaling in combination with analytical approaches can support the research on taste-influencing factors. In addition to the terpenoids known for their positive association with high sensory acceptance, acetophenone and styrene seem to be new candidates for a high acceptance perception in carrot. In contrast, low acceptance, characterized by bitter notes, astringency and off-flavor, correlated with a large range of volatiles and non-volatiles. Comparing the ingredient profiles of the individual cultivars, it is obvious that different qualitative and quantitative ingredient constellations of the cultivars can finally lead to similar sensory perceptions. According to the data obtained here, camphene, borneol, bornylacetate and myristicin, as well as falcarindiol, falcarindiol-3-acetate and two laserines, are among the most important key factors (off-flavors) for negative cultivar assessment.

## Figures and Tables

**Table 3:** Analytic data of main sugar compounds (%) in carrot root tissue. The plot mean and standard deviation (SD) for the individual cultivars as well as ANOVA (*p*) and Tukey’s significance test are given (different letters suggest significance *p* = 0.01, cultivars sorted by acceptance).

Cultivar	Fructose	Glucose	Sucrose
	Mean ± SD	Mean ± SD	Mean ± SD
Nevis	5.34 ± 0.14 de	5.80 ± 0.15 de	36.67 ± 0.44 ab
Himuro Fuyugosi Gosun	5.47 ± 0.22 de	5.40 ± 0.18 e	37.24 ± 0.70 a
Nantejska Polana	4.17 ± 0.09 f	3.79 ± 0.18 f	35.68 ± 3.56 abc
Nagykallo	5.18 ± 0.05 e	5.65 ± 0.10 de	35.40 ± 0.26 abc
Nantes Fancy	8.73 ± 0.20 a	9.23 ± 0.48 a	31.29 ± 0.64 ef
Nantes Liva	7.80 ± 0.29 b	8.34 ± 0.45 ab	33.31 ± 1.25 cde
Berlicumer Bercoro	7.92 ± 0.18 b	8.95 ± 0.20 a	31.89 ± 0.60 def
Hekinan Senko 5sun	5.99 ± 0.04 cd	7.23 ± 0.06 c	31.95 ± 0.18 def
Viking	5.52 ± 0.27 cde	6.30 ± 0.23 d	34.67 ± 0.59 bcd
Santa Cruz	5.72 ±0.19 cde	5.79 ± 0.18 de	35.32 ± 0.18 abc
Stratova	7.68 ± 0.44 b	8.33 ± 0.53 ab	33.66 ± 0.98 bcde
Beacon	7.29 ± 0.06 b	8.64 ± 0.07 ab	31.86 ± 0.31 def
Regulus Imperial	7.59 ± 0.14 b	7.99 ± 0.23 bc	29.25 ± 0.34 f
Vita Longa	6.20 ± 0.04 c	6.18 ± 0.16 de	37.13 ± 0.61 a
Vitaminaja	2.84 ± 0.03 g	2.74 ± 0.08 g	35.14 ± 1.28 abcd
Brasilia	7.65 ± 0.19 b	7.91 ± 0.10 bc	36.32 ± 0.56 abc
ANOVA (p)	0.00	0.00	0.00
Tukey p = 0.01			

**Table 4:** Parameters with a positive and negative influence on the sensory perception of carrot. Pairwise correlations between the sensory parameters and the volatile compounds, polyacetylenes and laserines, as well as sugars in the root tissue.

	GC	Acceptance	Sweetness	Aroma	Bitterness	Astringency	Off Flavor
sweetness		0.898					
aroma		0.975	0.952				
bitterness		−0.890	−0.774	−0.813			
astringency		−0.919	−0.706	−0.830	0.935		
off-flavor		−0.886	−0.644	−0.811	0.812	0.941	
α-pinene	B	0.473	0.532	0.554	−0.226	−0.292	−0.327
camphene	A	−0.458	−0.236	−0.366	0.488	0.593	0.718
hexanal	A	0.503	0.501	0.562	−0.324	−0.368	−0.340
β-pinene	B	0.459	0.454	0.439	−0.396	−0.395	−0.320
β-phellandrene	B	−0.207	−0.257	−0.145	0.490	0.284	0.051
styrene	B	0.617	0.464	0.591	−0.473	−0.639	0.712
o-cymene	A	−0.397	−0.314	−0.365	0.477	0.444	0.421
6-methyl-5-heptene-2-one	B	−0.568	−0.358	−0.441	0.700	0.653	0.583
hexanol	B	−0.553	−0.629	−0.553	0.565	0.424	0.298
γ-cadinene	A	−0.169	−0.206	−0.122	0.480	0.264	0.072
2-ethylhexanol	B	−0.345	−0.428	−0.318	0.436	0.341	0.260
bornylacetate	A	−0.513	−0.324	−0.463	0.436	0.593	0.772
β-caryophyllene	A	0.432	0.409	0.455	−0.279	−0.313	−0.393
borneol	A	−0.504	−0.307	−0.452	0.418	0.582	0.776
β-bisabolene	A	−0.288	−0.369	−203	0.444	0.301	0.272
acetophenone	B	0.600	0.494	0.614	−0.397	−0.530	−0.501
dextrocarvone	B	−0.594	−0.663	−0.591	0.669	0.544	0.521
β-ionon	A	−0.495	−0.309	−0.412	0.467	0.481	0.490
geranylacetone	A	−0.601	−0.496	−0.559	0.575	0.494	0.436
benzylalcohol	B	−0.526	−0.336	−0.430	0.637	0.638	0.664
4-methylphenol	B	−0.583	−0.496	−0.513	0.652	0.646	0.460
myristicine	B	−0.492	−0.288	−0.431	0.424	0.583	0.769
falcerindiol		−0.498	−0.335	−0.430	0.595	0.630	0.703
falcarinol-3-acetate		−0.680	−0.544	−0.638	0.705	0.720	0.736
laserin		−0.501	−0.288	−0.424	0.490	0.627	0.778
epilaserin		−0.527	−0.313	−0.455	0.485	0.635	0.794
sucrose		0.209	0.444	0.330	−0.067	−0.005	−0.058

GC-detection by HS-SPME (A) and SBSE (B) sampling technology; Positive correlations are displayed in blue, negative correlations in orange. The darkness of the colors corresponds to the level of correlation. Degrees of freedom = 14: P: 0.426 for α = 0.1, 0.497 for α = 0.05, 0.641 for α = 0.01, 0.742 for α = 0.001.

## Data Availability

Data are contained within the article and supplementary material.

## Supplementary Materials

The following supporting information can be downloaded: https://www.mdpi.com/article/10.3390/foods12244389/s1. Table S1: Plant material and origin; Table S2: Statistical data of the sensory analysis; Table S3: Statistical data of the volatile analysis (HS-SPME, SBSE) by ANOVA followed by Tukey’s test (*p* < 0.05); Table S4: Pearson correlation table of all sensory and analytical data (mean value based); Figure S1: Protocol of the hedonic acceptance test for carrot.
