# Supplementary material for: Sensory Perception and Consumer Acceptance of Carrot Cultivars Are Influenced by Their Metabolic Profiles for Volatile and Non-Volatile Organic Compounds
Source: Foods. 2023 Dec 6;12(24):4389. doi: 10.3390/foods12244389 (PMC10742604; doi:10.3390/foods12244389)
Supplement: Supplementary file 1 [file foods-12-04389-s001.zip › Figure S1 - Hedonic acceptance test for carrots.pdf]

Carrot – Sensory

Beaker-No. ....

Gender: w m   Age: .... Year   Profession: ....

Smoker: yes non   Hobby: ....

Please take of cover and eat carrot pices with the spoon.
Prevalence assess first, afterwards the charcaters. Please tick a box.

Acceptance

0	1	2	3	4	5	6

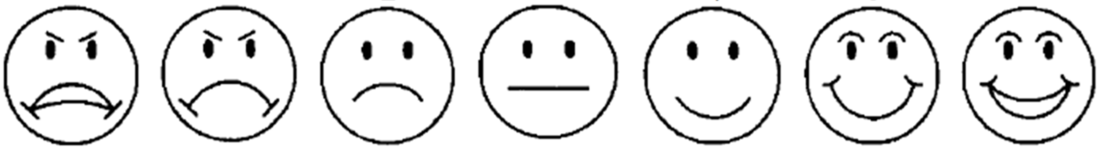

displeases me very	displeases me pretty much	displeases me something	neither nor	like me something	like me pretty much	like me very

Character

- sweet

☐ .... ☐ .... ☐ .... ☐ .... ☐ .... ☐ .... ☐
not at all — medium — intense

- bitter

☐ .... ☐ .... ☐ .... ☐ .... ☐ .... ☐ .... ☐
not at all — medium — intense

- astringent

☐ .... ☐ .... ☐ .... ☐ .... ☐ .... ☐ .... ☐
not at all — medium — intense

- aromatic as carrot

☐ .... ☐ .... ☐ .... ☐ .... ☐ .... ☐ .... ☐
not at all — medium — intense

- another aroma: ....

☐ .... ☐ .... ☐ .... ☐ .... ☐ .... ☐ .... ☐
not at all — medium — intense

Thank !
