# Supplementary material for: Sensory Perception and Consumer Acceptance of Carrot Cultivars Are Influenced by Their Metabolic Profiles for Volatile and Non-Volatile Organic Compounds
Source: Foods. 2023 Dec 6;12(24):4389. doi: 10.3390/foods12244389 (PMC10742604; doi:10.3390/foods12244389)
Supplement: Supplementary file 1 [file Table S2 Consumer acceptance study.pdf › Table S2 Consumer acceptance study.pdf]

Table S2 Consumer acceptance study of 16 carrot cultivars by 88 testers (cultivars sorted by acceptance)

	CV	Acceptance			Sweetness			Aroma			Bitterness			Astringence			Off-flavor		
		Mean	SD	Tukey	Mean	SD	Tukey	Mean	SD	Tukey	Mean	SD	Tukey	Mean	SD	Tukey	Mean	SD	Tukey
NE	Nevis	4,49	1,22	e	3,55	1,27	h	4,09	1,35	g	0,63	1,07	abc	0,40	0,87	a	0,34	0,95	a
HG	Himuro Fuyugosi Gosun	4,48	1,25	e	3,78	1,46	h	4,16	1,46	g	0,49	0,95	ab	0,28	0,73	a	0,41	1,00	a
NP	Nantejska Polana	4,22	1,14	de	3,43	1,33	gh	3,58	1,51	fg	0,38	0,68	a	0,37	0,82	a	0,40	1,04	a
NA	Nagykallo	4,10	1,15	de	3,16	1,30	fgh	3,44	1,30	fg	0,59	0,99	abc	0,37	0,71	a	0,39	0,94	a
NF	Nantes Fancy	3,70	1,17	d	2,95	1,38	efg	3,10	1,48	ef	0,49	0,89	ab	0,55	0,85	a	0,78	1,15	a
NL	Nantes Liva	2,77	1,29	c	1,89	1,30	ab	2,24	1,55	bcd	1,01	1,22	abc	0,68	1,08	ab	0,89	1,35	ab
HS	Hekinan Senko 5sun	2,74	1,74	c	2,60	1,52	def	2,44	1,69	cde	0,84	1,24	abc	0,94	1,15	bcd	1,64	1,90	cd
BB	Berlicumer Bercoro	2,67	1,25	c	1,91	1,41	bc	2,32	1,49	cd	0,97	1,28	abc	0,84	1,08	abc	1,02	1,52	ab
VI	Viking	2,61	1,67	c	2,52	1,45	def	2,60	1,54	de	1,56	1,67	abcd	1,52	1,72	d	1,77	1,90	abc
SC	Santa Cruz	2,55	1,56	c	2,66	1,54	def	2,50	1,60	cde	1,25	1,58	abcd	1,41	1,65	cd	1,66	2,02	abc
ST	Stratova	2,52	1,46	bc	2,03	1,53	bcd	2,40	1,58	cd	1,93	1,64	bcd	1,47	1,46	cd	1,53	1,76	abc
RI	Regulus Imperial	2,25	1,43	bc	1,72	1,40	ab	1,80	1,38	abc	1,48	1,52	abcd	1,27	1,48	bcd	1,74	1,98	abc
BE	Beacon	2,25	1,42	bc	2,24	1,29	bcd	2,26	1,39	bcd	1,66	1,74	abcd	1,29	1,33	bcd	1,53	1,76	abc
VL	Vita Longa	2,10	1,64	bc	2,40	1,59	cde	2,15	1,64	bcd	1,61	1,56	abcd	1,57	1,67	d	2,37	2,25	bc
VA	Vitaminaja	1,86	1,29	b	1,60	1,30	a	1,56	1,30	ab	1,53	1,49	d	1,24	1,55	bcd	1,70	1,79	abc
BR	Brasilia	0,90	1,56	a	1,68	1,64	a	1,13	1,45	a	2,06	1,77	cd	2,33	1,90	e	4,37	1,96	d
ANOVA	SQ	1413,18			659,72			996,55			589,45			453,09			1511,54		
	FG	15			15			15			15			15			15		
	MQ	94,21			43,98			66,44			39,30			30,21			100,77		
	F	47,97			21,70			30,10			4,47			17,61			10,12		
	p	0,000			0,000			0,000			0,000			0,000			0,000		
