# Supplementary material for: Sensory Perception and Consumer Acceptance of Carrot Cultivars Are Influenced by Their Metabolic Profiles for Volatile and Non-Volatile Organic Compounds
Source: Foods. 2023 Dec 6;12(24):4389. doi: 10.3390/foods12244389 (PMC10742604; doi:10.3390/foods12244389)
Supplement: Supplementary file 1 [file foods-12-04389-s001.zip › Table S1 - Material.pdf]

Table S1 Carrot plant material used in the sensory study and the cultivar type, the geographic origin as well as the breeding status

Code	Cultivar	Type[a]	Geographic origin[b]	Breeding status[c]
**NE**	Nevis	Nantes	EUR	F1
**HG**	Himuro Fuyugosi Gosun No.2	Kuroda	JAP	OP
**NL**	Nantes Liva	Nantes	EUR	OP
**ST**	Stratova	Berlikumer	RUS	OP
**VA**	Vitaminaja	Nantes	RUS	OP
**RI**	Regulus Imperial	Nantes/Flakkeer	EUR	OP
**HS**	Hekinan Senko 5sun	Kuroda	JAP	OP
**VK**	Viking	Chantenay	EUR	OP
**NA**	Nagykallo	Nantes	EUR	OP
**NP**	Nantejska Polana	Nantes	EUR	OP
**NF**	Nantes Fancy	Nantes	EUR	OP
**BB**	Berlicumer Bercoro	Berlikumer	EUR	OP
**BE**	Beacon	Nantes/Flakkeer	EUR	OP
**VL**	Vita Longa	Flakeer	EUR	OP
**SC**	Santa Cruz	Chantenay	USA	OP
**BR**	Brasilia	Flakkeer	BRA	OP

[a]Carrot root shape type, [b]Geographic origin of the breeders, [c]OP-open pollinated cultivar, F1 – hybrid cultivar
